# Condition-specific gene co-expression network mining identifies key pathways and regulators in the brain tissue of Alzheimer’s disease patients

**DOI:** 10.1186/s12920-018-0431-1

**Published:** 2018-12-31

**Authors:** Shunian Xiang, Zhi Huang, Tianfu Wang, Zhi Han, Christina Y. Yu, Dong Ni, Kun Huang, Jie Zhang

**Affiliations:** 1 Guangdong Key Laboratory for Biomedical Measurements and Ultrasound Imaging, School of Biomedical Engineering, Shenzhen University, Shenzhen, 518060 China; 2 Department of Medical & Molecular Genetics, Indiana University, Indianapolis, IN 46202 USA; 3 Department of Medicine, Indiana University, Indianapolis, IN 46202 USA; 4 Department of Electrical and Computer Engineering, Purdue University, West Lafayette, IN 47907 USA; 5 Department of Biomedical Informatics, The Ohio State University, Columbus, OH 43210 USA

**Keywords:** Alzheimer’s Disease, Co-expression, Condition-specific module, Bacterial and viral infectious pathway

## Abstract

**Background:**

Gene co-expression network (GCN) mining is a systematic approach to efficiently identify novel disease pathways, predict novel gene functions and search for potential disease biomarkers. However, few studies have systematically identified GCNs in multiple brain transcriptomic data of Alzheimer’s disease (AD) patients and looked for their specific functions.

**Methods:**

In this study, we first mined GCN modules from AD and normal brain samples in multiple datasets respectively; then identified gene modules that are specific to AD or normal samples; lastly, condition-specific modules with similar functional enrichments were merged and enriched differentially expressed upstream transcription factors were further examined for the AD/normal-specific modules.

**Results:**

We obtained 30 AD-specific modules which showed gain of correlation in AD samples and 31 normal-specific modules with loss of correlation in AD samples compared to normal ones, using the network mining tool lmQCM. Functional and pathway enrichment analysis not only confirmed known gene functional categories related to AD, but also identified novel regulatory factors and pathways. Remarkably, pathway analysis suggested that a variety of viral, bacteria, and parasitic infection pathways are activated in AD samples. Furthermore, upstream transcription factor analysis identified differentially expressed upstream regulators such as ZFHX3 for several modules, which can be potential driver genes for AD etiology and pathology.

**Conclusions:**

Through our state-of-the-art network-based approach, AD/normal-specific GCN modules were identified using multiple transcriptomic datasets from multiple regions of the brain. Bacterial and viral infectious disease related pathways are the most frequently enriched in modules across datasets. Transcription factor ZFHX3 was identified as a potential driver regulator targeting the infectious diseases pathways in AD-specific modules. Our results provided new direction to the mechanism of AD as well as new candidates for drug targets.

**Electronic supplementary material:**

The online version of this article (10.1186/s12920-018-0431-1) contains supplementary material, which is available to authorized users.

## Background

Alzheimer’s disease (AD) is a slow progressing neurodegenerative disease, affecting about 40 million people worldwide today. More than a century has passed since AD was first identified, but the disease mechanism still remains unclear. Till today, no curative treatment is available for AD [1]. The prevalent AD hypothesis is the “amyloid cascade” hypothesis [1] proposed 25 years ago. This hypothesis suggests that the abnormal accumulation of insoluble β-amyloid (Aβ) peptide in cerebral plaques leads to neurofibrillary tangles (NFT) by hyper-phosphorylated tau protein, which then triggers downstream inflammation response, synapse loss, neuron death, and dementia. Although Aβ and NFT are the two most prominent neuropathologic features of AD, they are not unique to AD, and it is still not clear whether they are the causes or the results of AD or if there is causal relationship between the two [2]. AD-specific pathways, biological processes, and driver genes remain to be found.

Since AD is a complex disease involving multiple biological processes, a systems biology approach is needed to identify key pathways and genes for the development of AD. Network-based approaches are commonly adopted in systems biology [3]. In this paper, we apply a co-expression network analysis on multiple transcriptomic datasets of AD and normal brain samples to identify biological processes and potential driver or regulatory genes specifically associated with or disrupted in AD. Currently, most AD transcriptomic analysis studies are focusing on identifying differential expressed genes from various brain regions of AD patients [4, 5], and there are only few work studied highly correlated pairs of genes to obtain gain or loss of co-expression in AD [6].

The network mining algorithm used here is lmQCM [7], which was developed by us to identify condition-specific gene co-expression network (GCN) modules as a whole in brains of AD patients as compared to normal controls and look for potential “driver” regulators for AD. lmQCM has been previously applied to disease-specific network mining in several studies, and identified frequently co-expressed modules in pan-cancer scale as well as in specific cancer types and other diseases [8–10]. Unlike the widely used WGCNA algorithm, which is based on hierarchical clustering, lmQCM allows modules to overlap with each other and be capable of identifying smaller local gene modules often induced by copy number variants [7]. As a result, we identified 61 gene modules of distinct functional categories with gain or loss of correlations in AD samples, many of which have been linked to AD pathology while other are new for AD. Remarkably, we found 9 enrichment terms pertaining to infectious diseases in AD-specific modules while the tight junction pathway was detected for a normal-specific module, which supports the hypothesis that brain infection may be the causes for AD. Moreover, we conducted transcription factor analysis of the condition-specific modules and discovered differentially expressed upstream regulators for 16 gene modules that are specific to AD or normal. Specifically, we identified ZFHX3 as a key regulator for multiple infectious diseases pathways which are highly enriched in AD-specific modules. This study made exciting discoveries of potential new AD candidate driver genes and underlying pathways, therefore offers new insights and directions on mechanism and drug design for AD.

## Methods

### Data and sample pre-processing

Two large microarray datasets GSE5281 [5] and GSE48350 [6] from the NCBI Gene Expression Omnibus (GEO) containing multiple regions of AD and normal brains were downloaded, each with over 20 samples for each specific brain region in each condition. Both datasets were generated using Affymetrix HU133 2.0 Plus platform. An RNA-seq dataset was also obtained with transcriptome-wide FPKM values for AD and normal samples of multiple brain regions from the Allen Brain Institute (http://aging.brain-map.org). In total, we processed 500 samples from 10 different brain tissues, of which 197 samples are from 111 AD patients and 303 samples are from 97 healthy normal persons. The sample and dataset details are shown in Table 1. The two microarray datasets were processed using R/Bioconductor package *Affy* [8] to generate normalized expression values by RMA normalization using their default parameters. All datasets were pre-filtered to remove probes without gene annotation, while for genes with multiple probes, we followed the same procedure as in previous studies [10, 11] to select the probeset with the highest mean expression value. Only samples from patients diagnosed as “AD” or “Probable AD” were considered as AD samples. Genes with more than 50% zero expression levels across samples of AD or normal were removed from all datasets. For the RNA-seq dataset, we kept genes with FPKM value larger than 1 in at least one sample. Before constructing the co-expression network, all genes with variance in the bottom 20% percentile of the entire transcriptome were discarded. The FPKM values of the filtered genes were log_2_-transformed using log_2_(FPKM + offset) with an offset = 1.0. After the filtering, we obtained expression levels of 17,547 genes for GSE5281, 16,686 genes for GSE48350 and 18,789 genes for the Allen Brain Institute dataset. Since the samples are post-mortem samples from confirmed AD patients with pathological changes well-spread in the brain, we aim to search for the gene modules commonly presented in all of the AD-affected brain regions, therefore, we combined samples from all brain regions for module mining. For each dataset, t-test was used to identify the differentially expressed genes with a cut-off of statistical significance *p*-value <0.05 and foldchange >1.5.

**Table 1:** Summrize of datasets we used in the analysis

Dataset	Regions	Total samples	AD samples	AD patients	Normal samples	Normal persons	Subjects per person
GSE5281	6	161	87	23	74	13	1~12
GSE48350	4	253	80	58	173	28	1~8
Allen brain	4	86	30	30	56	56	1

### Gene co-expression network (GCN) construction and module detection

We performed lmQCM network mining on each of the 3 pre-processed datasets separately. First, AD and normal samples were separated into different groups within each dataset. Next, Pearson correlation coefficient (PCC) between each pair of genes were calculated for AD and normal samples separately. As a result, we obtained weighted co-expression networks for AD and normal samples respectively for each dataset, in which the nodes are genes and the weights of the edges are PCC values. Next, local maximized Quasi-Clique Merger (lmQCM) previously developed by our group was applied to identify tightly co-expressed gene modules in the weighted network [7]. It has been previously applied for biomarker prediction in multiple types of diseases including colon, breast, lung cancers, leukemia, and Parkinson’s disease as well as disease gene discovery [9–12]. The parameters for lmQCM were set as follows: *t* = 1.0, lambda =1.0, gamma =0.3, beta =0.3,minimum cluster size =10. The R package for this network mining tool is available in CRAN as “lmQCM”, and the web-version is available as well (https://apps.medgen.iupui.edu/rsc/tsunami/).

### Comparison of modules detected by different GCN algorithms

To compare our method with commonly used WGCNA, we applied WGCNA to our datasets for GCN construction and module detection. We compared each module identified by lmQCM to modules detected by WGCNA with the same dataset. For each module identified by our method, we obtained one matched module in WGCNA modules, which showed the most gene overlapping. The ratio of genes overlapped with matched WGCNA module was calculated for each lmQCM module.

### Test the robustness of lmQCM algorithm with Gaussian noise simulated data

We tested the robustness of the lmQCM mining algorithm in noisy data. For each dataset, we first introduced additional 5, 10 and 15% of random Gaussian noise into standardized expression data matrix (zero mean and unit variance). Next, lmQCM modules were mined with the same parameters as described previously for the same three datasets with noise. The modules identified before and after adding noise data were compared for consistency by evaluating gene overlaps between experiments. For each module, the ratios of overlapped genes to original modules were calculated respectively in three noise levels. Boxplots were generated for overlapping ratios for the results from three noise level as compared to original modules. Some modules may be exactly the same before and after adding noise data, and these modules were counted.

### Compare modules between AD and normal samples to obtain condition-specific GCN modules

In order to determine if a gene module is condition specific, for each module detected in a specific condition (AD or normal), we examined if the expression profiles of genes in that module are significantly correlated in one condition but not in the other with the previously developed metric Centered Concordance Index (CCI) [12]. CCI values range from 0 to 1, which indicate the extent of overall correlation of genes in a module. Larger CCI values imply more densely correlated genes in that module. We focused on the modules whose CCIs are significantly high in one condition (after multiple-test compensation) while not in the other. For a module containing n genes, we randomly choose n genes from expression matrix and calculate the CCI. This procedure was repeated 1000 times to obtain the CCI distribution. The z-score (Z_CCI_) for the testing module CCI based on the random sampling was calculated. This gives a measurement on how significant is the observed CCI for the tested gene module in the background of entire genome. For each gene module in AD or normal samples, we calculated two CCIs, using the expression data from AD samples and normal samples separately, and the Z_CCI_ are calculated for each condition. Gene modules that are significant (Z_CCI_ ≤*τ*) in one condition but not significant (Z_CCI_ >*τ*) in the other are considered as condition-specific modules. The threshold *τ* is determined based on the significance requirement that *τ* is chosen such that the one-tail *p*-value for the Z_CCI_ is less than 0.05 for a specific gene module. Additionally, certain modules contain z-scores of opposite signs, which means the modules gain correlation in one condition while losing correlation in the other. For such cases, although *p*-values are less than 0.05 in both conditions, they are included for downstream analysis due to the opposite change of correlations.

### Functional enrichment analysis

**Fig. 1:**
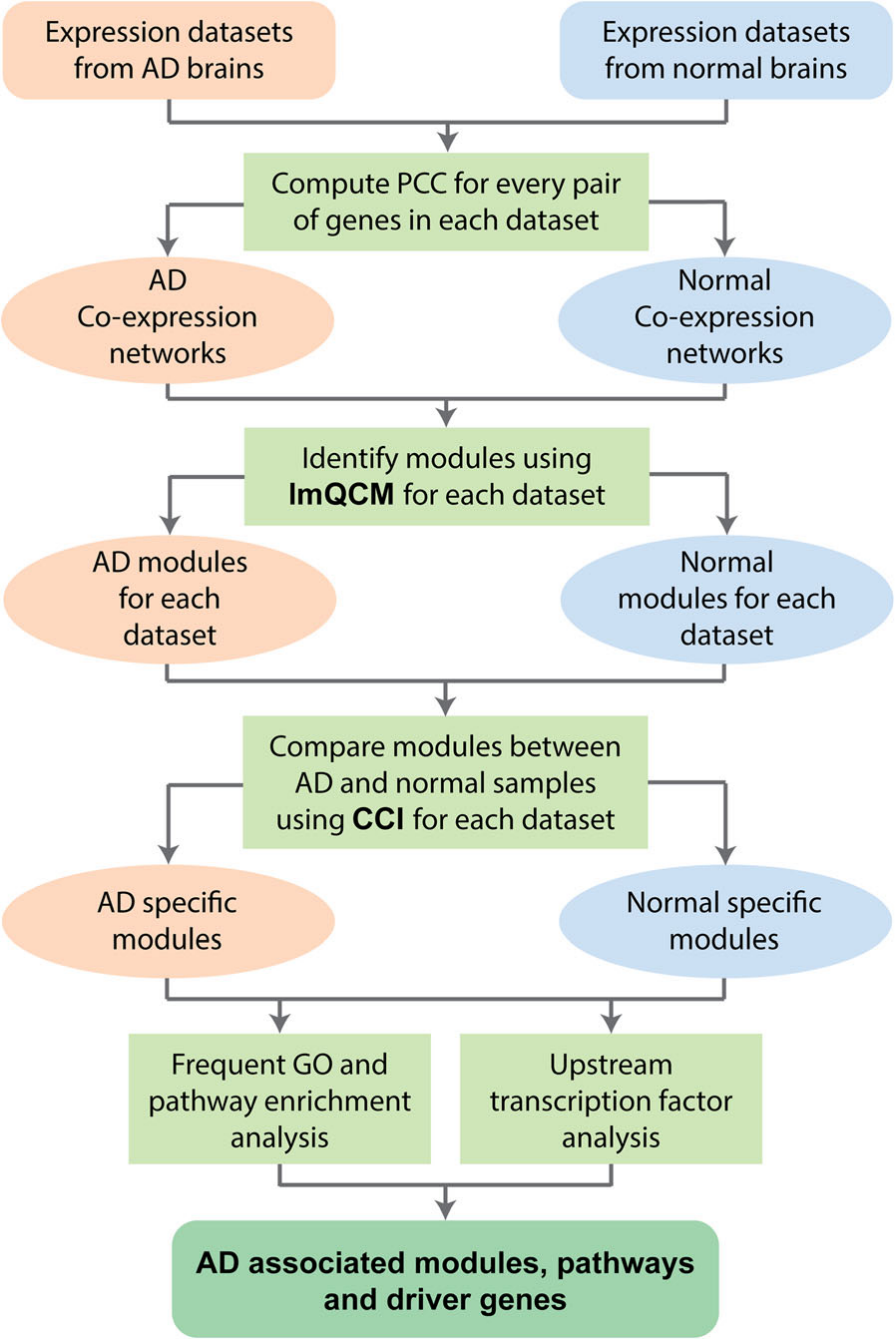
Workflow to identify condition-specific co-expression modules and AD associated pathways and driver genes

The R package Enrichr [13] was used to perform gene ontology (GO) and pathway enrichment analysis of the module genes identified from each of the three datasets. “GO_Biological_Process_2017b” (BP) and “KEGG_2016” databases were used. Only GO BP terms or KEGG pathway with enrichment p-value less than 0.01 were considered significant enriched. Next, the frequencies of specific GO/pathway terms were counted for AD and normal specific modules respectively. Only GO terms appeared in at least two of the three datasets were included for further study. Redundant Gene Ontology terms were merged by REViGO [14]. The workflow of the entire analysis is shown in Fig. 1.

### Upstream regulators identification

To search for upstream regulators of a module, Enrichr [13] was used with the “TRANSFAC_and_JASPAR_PWMs” database. The analysis with the database returns the transcription factors (TFs) that regulate genes in the modules (*p*-value <0.01). The frequencies of enriched TFs in AD specific or normal specific modules are also counted. Student’s t-test (cutoff foldchange>1.5, p-value <0.05) was used to check whether these transcription factors are differentially expressed between AD and normal samples. Enriched TFs that differentially expressed were retained as upstream regulators of the modules. To further investigate the exact enriched pathways or GO biological processes of the modules affected by the differentially expressed TFs, we compared the targets of specific TFs that occurred in the modules and its enriched functional term members. TFs with more than two shared targets and enriched functional term members of certain module are highlighted as most significant upstream regulators.

## Results

### GCN modules specific to AD or normal brain tissues

We obtained 101 densely GCN modules from AD samples and 77 modules from normal samples in three datasets using the workflow (Fig. 1). To compare the modules identified by our method with the popularly used WGCNA [15], we also applied WGCNA to the same datasets in our work to mine densely correlated modules. Number of modules and module size range are listed in Table 2 for both lmQCM and WGCNA method. From the table, we can see that our method identifies more modules with smaller sizes than WGCNA. For example, for GSE48350 AD dataset, WGCNA returned 22 modules while our method gets 49 modules. The module size range of WGCNA is 33~3567 while the modules identified by our method ranges from 10~391. To check if the genes in module identified by lmQCM are consistent with WGCNA result, we compared the genes in each module to modules of WGCNA. For example, in GSE48350 AD dataset, for each module identified in GSE48350 AD dataset by our method, we see a matched module in WGCNA which showed the most gene overlapping. Over 73% of modules (36/49) of our method shared over half of gene members with matched WGCNA modules (Additional file 1: Table S1), which indicates that the GCN modules from lmQCM are consistent with the ones from WGCNA but tighter and densely connected, often implying more specifically enriched in biological processes.

**Table 2:** Number of modules identified in three datasets and size range of the modules

Dataset	Module number	Module size range	Module number	Module size range
Method	lmQCM	lmQCM	WGCNA	WGCNA
GSE5281_AD	26	10~275	13	101~5438
GSE48350_AD	49	10~391	22	33~3567
Allen Brain_AD	26	10~145	29	34~3466
GSE5281_control	31	10~528	25	35~2657
GSE48350_control	32	10~176	20	34~3870
Allen Brain_control	14	10~106	13	40~4778

We applied data pre-filtering to remove most of the noise before module mining. However, to ensure our lmQCM algorithm is robust under noisy condition, we tested lmQCM robustness by introducing 5, 10 and 15% of Gaussian random noise to the expression data before applying lmQCM for module mining. Ratios of overlaps between 178 original modules and modules obtained after noise addition showed that the same or highly overlapped modules were detected with even 15% of Gaussian noise (Additional file 2: Figure S1). The average overlapping ratios are high (93.48, 88.83, and 85.31% for 5, 10, and 15% of noise, respectively). In particular, among 178 modules, 112, 78, and 72 of them are exactly the same when introducing 5, 10, and 15% of noise. These results demonstrate that lmQCM algorithm is very robust under noisy condition.

**Fig. 2:**
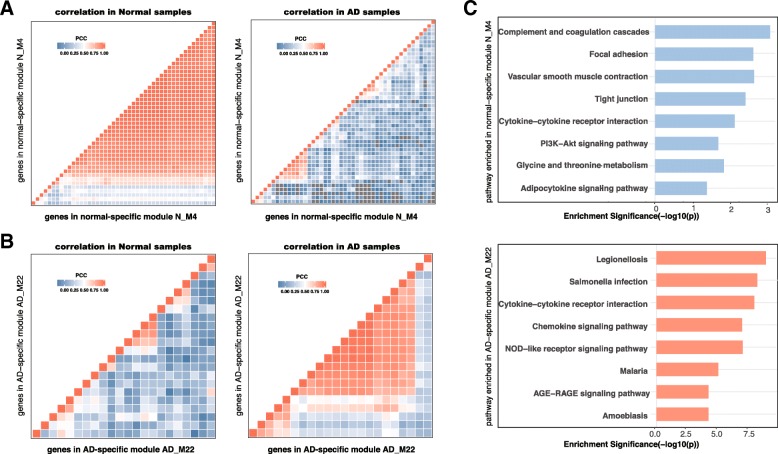
Correlation heatmap of two example condition-specific modules and matched enriched pathway analysis of each module. **a** Correlation of gene pairs in normal-specific N_M4 in normal samples (left) and in AD samples (right) **b** Correlation of gene pairs in AD-specific AD_M22 in normal samples (left) and AD samples (right). **c** Top enriched pathways in normal-specific and AD-specific modules

Centered Concordance Index (CCI) [15] was used to quantify the gain or loss of co-expression in AD vs, normal brains, for each module detected in a specific condition (i.e. AD or normal). First, we calculated CCI in both AD and normal expression groups. Z-scores of CCI (Z_CCI_) between the two conditions followed by multiple-test compensation was used to determine if the expression profiles of genes in the module are significantly correlated in one condition but not in the other (see Methods for details). This resulted in 30 AD specific modules (AD_M1-AD_M30) and 31 normal specific modules (N_M1-N_M31) for three datasets (see Additional file 3: Table S2). The AD-specific modules showed gain of connectivity or enhanced coregulation between genes in AD samples and the normal specific modules showed loss of connectivity or reduced coregulation between genes in the module in AD samples (Fig. 2a and b). The remaining 117 modules were assumed to perform conserved functions across the AD and normal conditions, therefore, we focused on the condition-specific modules in the following analysis.

### Frequent functional enrichment analysis of the condition-specific GCN modules revealed functions associated with AD pathology

The densely correlated modules are likely to be co-regulated and perform similar functions. Genes in AD specific modules gain correlation in AD relative to normal condition while normal specific modules loss correlation in AD as compared to normal condition. These gene co-expression pattern change can be the indication of the module genes functional change, which potentially contributes to AD etiology and pathology. Therefore, we conducted GO biological process and pathway enrichment analysis for the 30 AD-specific modules and 31 normal-specific modules. Figure 2 showed the gene expression correlation changes and enriched pathways of an example normal-specific module N_M4 from GSE4830 dataset, and AD-specific module AD_M22 from GSE5281 dataset. Enriched pathways for module N_M4 are complement and coagulation cascades, focal adhesion, vascular smooth muscle contraction, tight junction, and cytokine-cytokine receptor interaction. In AD-specific AD_M22, genes are enriched in legionellosis, TNF signaling pathway, salmonella infection, chemokine signaling pathway, NOD-like receptor signaling pathway, malaria, AGE-RAGE signaling pathway, and amoebiasis. All significant enriched GO terms and pathways are summarized in Additional file 4.

**Fig. 3:**
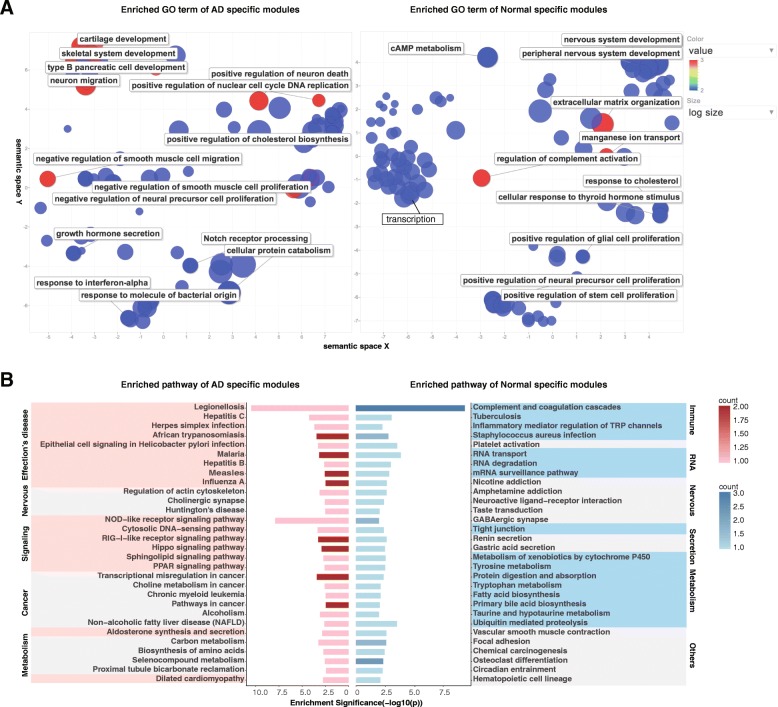
Frequent GO and pathway enrichment analysis of AD-specific modules and normal-specific modules. **a** Top enriched GO term of AD-specific modules (left) and top enriched GO terms of normal-specific modules (right). The value is the frequency of the term occurred in modules from three datasets. The size indicates the number of genes in a specific term. **b** Top 30 enriched pathways of the modules, while the counts are the frequency of a term occurred in the modules from three datasets which reflected by the red/blue color bar. The pink/blue/grey shading in the pathway list separates the pathways into different categories and summarizes them on the left/right side

Since the modules are from three independent transcriptomic datasets each with different brain region compositions, functional enrichment terms that occurred across modules from three datasets are more possible to be prevalent to AD. Therefore, instead of checking all GO terms of the modules, we focused on GO terms that were significantly enriched in modules from at least two datasets as frequently enriched GO terms. As a result, we obtained 257 frequent enriched GO terms (Enrichr *p*-value <0.01) for AD-specific modules and 162 such terms for normal-specific modules. We further merged similar GO terms with the REVIGO online tool [14]. In general, in both AD and normal-specific modules, we found distinct GO BP terms to each condition. As shown in Fig. 3a, most frequently enriched GO BP terms in AD-specific modules include response to interferon-alpha,response to molecule of bacterial origin,regulation of neuron death, negative regulation of neural precursor cell proliferation, neuron migration, cartilage development, skeletal system development, and mitochondrial protein processing. The normal specific modules are enriched for genes involved in nervous system development, synapse assembly, regulation of complement activation, transcription associated regulation processes, and cell proliferation associated processes. Many of these biological processes have previously been linked to AD-related changes [16–21]. For example, Yokota et al. [18] identified the same enriched GO biological processes about negative regulation of gene expression. Zhang et al. [21] reported AD associated modules share enriched GO BP of extracellular matrix, nervous system development, synaptic transmission, and neurotrophin signaling. Some of our enriched GO terms are more specific, such as response to interferon-alpha, response to molecule of bacterial origin, which is similar but more specific to immune response reported in [21].Additionally, some of the enriched GO biological processed identified here are novel, such as cartilage development and skeletal system development, which may infer potential new mechanisms of AD pathology.

As for the KEGG pathway analysis, we obtained 60 enriched pathways in AD-specific modules and 47 in normal-specific ones (Enrichr *p*-value <0.01). Among the enriched pathways, 16 are common between AD-specific modules and normal-specific modules, while the remaining are specific to either AD or normal modules. We focused on the condition-specific pathways in the following analysis. Certain enriched pathways frequently occurr in the modules across three datasets as well, so we computed the frequency of enriched pathway terms in modules.

As shown in Fig. 3b, the AD-specific pathways include metabolic associated pathways, bacterial and virus infections, cancer associated pathways, neuron associated pathway, Hormone, various signaling pathway, PPAR signaling pathway, regulation of actin cytoskeleton, and non-alcoholic fatty liver disease (NAFLD). Remarkably, although several previous studies have inferred immune associated pathway such as immune response and microglia pathway in AD samples [17, 21–23], we first identified the specific infections pathway, termed Influenza A, Measles, Hepatitis C, Herpes simplex infection, and RIG-I-like receptor signaling pathway for AD specific modules. The normal-specific pathways include GABAergic synapse and neuroactive ligand-receptor interaction, amino acid metabolism, complement and coagulation cascades, tight junction, platelet activation, renin secretion, and RNA metabolism pathways. Among which the tight junction, platelet activation and renin secretion pathways are first identified compared to previous co-expression analysis of AD samples [17, 18, 21]. The more specific terms identified confirmed that our method is able to discover more locally densely correlated modules.

Pathways enriched in AD-specific modules that have not been previously related to AD may represent novel disease mechanisms and processes, which include, for example, phospholipase D signaling pathway and osteoclast differentiation. Moreover, the comprehensive representation of gene-gene interactions in the already known AD-associated pathways can uncover novel gene members, thus allowing us to examine known pathologic mechanisms in more details.

Among these pathways, the most conspicuous ones in AD-specific modules are infectious disease pathways. Infectious disease pathways are identified in AD-specific modules from all three independent datasets, including module AD_M9 in dataset GSE5281, module AD_M22 in GSE48350 and module AD_M25 in the Allen Brain dataset. The enriched infectious disease pathways include bacterial infections such as African trypanosomiasis, legionellosis, salmonella infection, parasitic infections like malaria, and viral infections like Influenza A and Hepatitis C. In normal-specific modules, the enriched tight junction pathway in module N_M4 caught our attention. The genes in the module that occurred in tight junction pathways are RAB13, MYH11, MYL9, and YBX3. Genes in the module enriched in tight junction are tightly correlated in normal brain samples, where such correlation is lost in AD brain samples. It suggests that the function of tight junction may get disrupted in AD brains thus provide more access of virus, bacteria, and even parasites into the brain.

### Upstream transcription factor analysis for the infectious disease pathways leading to discovery of ZFHX3 as a potential driver regulator

To understand if there are key regulators for the biological processes and pathways discussed above in AD, we searched for upstream regulators among the modules by performing regulatory transcription factor analysis to identify gene interactions and regulatory elements within each module, again using the Enrichr [13] package. Since co-expression relationship is often resulted from co-regulation, the pursuit of upstream regulators for the condition specific GCN modules can lead to new insights on the potential driver genes for AD or related symptoms.

As a result, 15 of 30 AD-specific modules and 22 of 31 normal specific ones were found to have enriched upstream transcription factors (TFs). We then checked whether these TFs are differentially expressed between AD and normal samples. As a result, six AD-specific modules enriched with eleven differentially expressed TFs and ten normal-specific modules with 9 differentially expressed TFs were identified, the details are shown in Tables 3 and 4. Here we observed that AD-specific module AD_M1, module AD_M14, and normal-specific module N_M9 are targeted by multiple TFs while three TFs termed BCL6, JUND, and TCF4 are enriched in two different normal-specific modules.

**Table 3:** Transcription factors enriched in AD-specific modules that showed differentially expressed between AD and normal brain samples

Differentially expressed TFs	Module name	TF target genes in the module
FOS	AD_M5	MED12;IL11;GPR34;DOCK8;MRC1;PLA2G7
JUN	AD_M3	FBXW4;NOTCH4;HDAC11;PLEKHB1;ICAM2;CABLES1;WDR83;GAB2;PTH1R;FGF1;AIF1L;TGFBR2;TM7SF2;KIAA1598;GNA12;PROM1;KANK3;BOK;TBC1D16
SP1	AD_M1	DDR1;SLC27A1;LEPROT;MAOA;HDAC11;NDRG2;TRIM8;LFNG;CCND3;ZNRF3;NACC2;SH3PXD2B;UAP1L1;PRDM16;LRIG1;PLCG1;ARHGEF40;NEO1;RREB1;TBC1D16;NKX2–2;MYO10;PAX6;GRIN2C;PRDX6;ERBB2IP;ZFP41;RFX4;DLC1;PPP1R1B;PLXNB1;PLEKHO2;PPARA;MEGF8;EZR;FGFR3
ZFHX3	AD_M25	FAM122B;RSAD2;OAS1;SAMD9;TRIM21;USP18;IFIT3
ZNF281	AD_M1	DDR1;PPP1R14B;ADCYAP1R1;PHLPP1;SLC44A2;MAOA;WFS1;PBXIP1;NDRG2;TRIM8;ERBB2IP;TUBB2B;DLC1;TRPS1;NACC2;PPP1R1B;PRDM16;ITGA6;PLEKHO2;NEO1;RREB1;GPR125;RAPGEF3
TEAD4	AD_M1	ADCYAP1R1;LEPROT;MAOA;AQP4;NDRG2;NPAS3;PHF21B;LFNG;SOX2;CCND3;SLC25A29;ZNRF3;GNA12;SH3PXD2B;UAP1L1;PLCG1;SOX9;CCDC77;ARHGEF40;NEO1;RREB1;
		NKX2–2;SNTA1;PAQR6;YES1;MYO10;WFS1;C17ORF62;GRIN2C;PRDX6;PLEKHA7;GADD45G;KIAA1755;ZFP41;FAM195B;RFX4;DLC1;PPP1R1B;PLEKHO2;PPARA;MEGF8;EZR
MIB2	AD_M1	SLC27A1;ALDH1L1;COL16A1;TTYH1;PON2;MED12L;EGFR;LFNG;SLC25A29;HEPH;PERP;NACC2;SH3PXD2B;PHACTR3;S100A13;KCNN3;ACSS1;ARHGEF40;PAMR1;RREB1;STOX1;ZNF462;PPP1R14B;SERPINB1;PAX6;C17ORF62;PLEKHA7;AIF1L;ZFP41;DLC1;PMP22;MEGF8;SFXN5;ITM2C
MEF2A	AD_M12	NR4A2;TNMD;GPR64;TMEM74;SAMD3;TTN
PCBP1	AD_M1	DDR1;SLC27A1;LEPROT;SDC4;MAOA;AK4;NDRG2;NPAS3;CCND3;ZNRF3;NACC2;SH3PXD2B;PRDM16;UAP1L1;LRIG1;PLCG1;ARHGEF40;NEO1;RREB1;CD99;TBC1D16;TMEM184B;GPT2;PAX6;GRIN2C;PRDX6;ERBB2IP;GLUD1;FAM195B;ZFP41;DLC1;PLXNB1;PLEKHO2;PPARA;MEGF8;EZR;FGFR3;ZNF652
SMARCA2	AD_M14	FOXC2;SLC38A1;FOXD2;CRABP2;LRP1;CCDC25;PLA2G2A;KCTD9;KLF5;OS9;PPDPF;P4HB;SLC22A8;PHLDB2;PTGDR
STAT1	AD_M14	DSP;CLIC3;CRABP2;LRP1;PRDM6;CXCR4;BMP4;COL3A1;COL1A2;PDLIM2;MLPH;NOV;SLPI;SPTLC3;FRZB;PPDPF;SLC26A7

**Table 4:** Transcription factors enriched in normal-specific modules that showed differentially expressed between AD and normal brain samples

Differentially expressed TFs	Module name	TF target genes in the module
BCL6	N_M9	CSRNP3;RBFOX2;UBE2R2;THRAP3;NRXN1;OTUD7A;FNIP2
BCL6	N_M13	TRPC3;RSPO2;BTBD11;ITM2A;HS3ST1
JUND	N_M9	GABRA1;CSRNP3;UBE2R2;CCNY;NRXN1;GTPBP8;RUNX1T1
JUND	N_M30	CFH;C1S;MS4A4A;TNFSF13B
REPIN1	N_M9	MYLIP;RBFOX2;CCNY;RAB14;GABRA4;KLHL8;KIF1A;SRCIN1
CBFB	N_M11	DSP;CAMK2D;PTGER3;ARHGAP6
TCF4	N_M12	CLEC3B;CD163;COL1A2;SLC13A4;C7;C1S;ADH1B;OGN;FBLN1;CD14;CES1
TCF4	N_M24	GSTM3;SLC13A4;C2ORF40;LRRC18;GOLM1;KCNJ13;SERPINF1;TYRP1;ABCA4;BUB1B;HTR2C;FBLN1;SULF1;CLDN2;PRLR;LOXL1;GHR;SLCO1B3;SOSTDC1;OTX2;HPD
EGR1	N_M15	WWC3;CORO6;UPP1;FRS3
SOX10	N_M23	RGS2;ALKBH5;SNX16;BDNF;HS3ST2
ZBTB7A	N_M25	EGR2;NR4A3;FOSB;SIK1;FOS;JUNB
APEX1	N_M28	IGBP1;CLCN4;PHF20;UBE2D3;ZNF12

To further investigate the exact enriched pathways or GO BP of the modules affected by the differentially expressed TFs, we examined the targets of specific TFs and their regulated pathways that are enriched in our GCN modules (see Additional file 5). Among them, AD-specific module AD_M1 is regulated by SP1, TEAD4, PCBP1 with targets in the pathway of regulation of actin cytoskeleton, cAMP signaling pathway, PI3K-Akt signaling pathway, metabolic pathways, Alcoholism, and PPAR signalling pathway. AD-specific module AD_M3 is regulated by JUN, which target genes enriched in the pathway in cancer. AD-specific module AD_M25 is regulated by ZFHX3, with target genes in infectious disease pathways. For normal specific modules, JUND targets module N_M9 which is enriched in GABAergic synapse, while TCF4 targets genes both in enriched platelet activation pathway of module N_M12 and enriched neuroactive ligand-receptor interaction pathway of module N_M24. ZBTB7A target genes in module N_M25 with respect to osteoclast differentiation pathway.

**Fig. 4:**
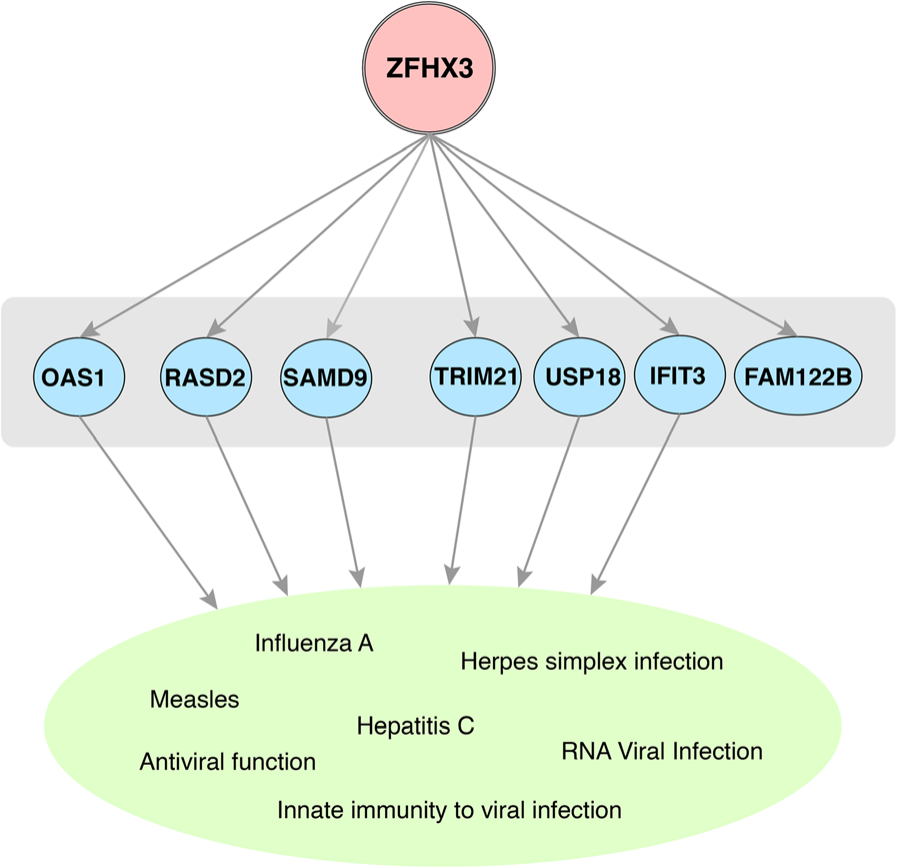
The key upstream regulator identified by transcription factor analysis. ZFHX3 targets seven genes in module AD_M25. Most of the genes in that module are associated with infectious disease, indicating the ZFHX3 as a key regulator of the module

What caught our interest is the transcription factor ZFHX3 that targets AD-specific module AD_M25 which are associated with infectious diseases from our enrichment analysis. ZFHX3 is up-regulated in AD vs. normal samples. The up-regulated TF ZFHX3 targets seven genes in module AD_M25, where two genes OAS1 and RSAD2 are detected in infection pathways, and the other five genes FAM122B, SAMD9, TRIM21, USP18 and IFIT3 are also known to be related to infectious disease (Fig. 4). The genes OAS1 and RSAD2 play important roles in infection pathway, imposing an activation effect on several infectious disease response pathways detected in AD compared to normal samples. As for the other genes, SAMD9 has been reported as an innate host antiviral stress response element that participates in the formation of antiviral granules [24]; TRIM21 was reported to promote response to viral infections [25]; USP18 plays a role in innate immunity to viral infection [26, 27]; IFIT3 also involved in antiviral functions according to previous research [28, 29], and FAM122B is new here to be associated with infections. Previous research showed that genetic variants at ZFHX3 is related to dementia [30]. In summary, the new results provide exciting convergent evidences for the specific infection responses activated in AD. The potential driver regulator roles of these pathways, particularly ZFH3, should be further studied in AD.

## Discussion

Genes in a co-expressed module share similar (i.e. correlated) expression profiles in certain conditions and they are often co-regulated by the same set of regulators (e.g. transcription factors) or residing on proximal regions on the chromosome. In addition, they often participate in related biological processes. Thus, mining GCNs can lead to discoveries in novel gene functions, protein-protein interactions (PPI), key genetic regulators for diseases and biological processes, functional structural variations, and disease biomarkers. More importantly, by identifying condition specific GCN modules, we can identify potential “driver” regulators for AD. Here we took advantage of the large amount of publicly available transcriptome data from human AD studies and applied our network mining approach to identify condition-specific GCN modules associated with AD. GCN modules in AD have been studied previously. In Dua et al. [31], network analysis of hippocampal gene expression data of 22 AD patients showed enrichment of viral genome expression, glycogen catabolic process, triglyceride metabolic process, cell death, and alcohol metabolic process. In Xia et al. [32], by combining differential expression analysis and GCN analysis, processes such as increased oxidative stress, along with alterations in lipid metabolism in neurons have been suggested to be associated with AD pathology. In Ding et al. [33], an integrated approach based on multi-data fusion on AD with the consideration of TF on the target gene regulation led to discovery of transcription factors E2F4 and ATF1 as well as immunoregulatory and neurogenesis processes in AD pathology. In comparison, our analysis involved much more samples, brain regions (see Table 1) and three independent datasets. Besides AD samples, we included normal samples in our analysis and identified condition-specific modules, which are unique and differ from those three works. The condition-specific modules reveal gain or loss correlation in AD compared to normal samples. By linking the modules to its enriched biological processes or pathways, we delineated pathways and gene targets causally related to AD pathology in many respects. Our results share some consistency with previous findings, such as immune response related processes, but with more details on the infectious pathways and potential regulators.

Many of the enriched GO terms for AD-specific modules have previously been reported to be associated with AD [34–37]. For instance, the enrichment of regulation of neuron death [38], negative regulation of neural precursor cell proliferation [39], and neuron migration [20] may explain the neuronal death characteristic of AD. The enrichment of mitochondrial protein processing is no surprise either given that neurons rely heavily on the functions of mitochondria and many research results showed that dysfunction in mitochondria processes are heavily involved in AD pathogenesis [40, 41].

The most interesting findings are the infectious diseases pathways, which are detected in all three datasets. Other pathways that have been implicated in AD are PPAR signaling pathway, regulation of actin cytoskeleton, Non-alcoholic fatty liver disease (NAFLD), and several signaling pathways. In particular, enriched pathways in cancer are frequently detected in AD-specific modules among three datasets, and as reported, there is an inverse relationship between cancers and AD [42, 43]. The biological processes newly identified in this work that are not previously associated with AD are cartilage development and skeletal system development, suggesting new insight and hypothesis related to AD development.

The enriched biological processes and pathways in normal specific GCN modules, which are disrupted in AD samples, varies substantially. Besides nervous system development, synapse assembly, transcription, and cell proliferation associated biological processes, GABAergic synapse and neuroactive ligand-receptor interaction pathways are also disrupted in AD, which all fit the neuron degeneration characteristic of AD well. Other pathways that have effects on AD like tight junction [44–46] and platelet activation [34] are also identified. Interestingly, genes involved in tight junctions are only identified in normal-specific modules (see Additional file 3), which indicates that the dis-concordance of gene interaction in AD may contribute to the loss of tight junctions in blood brain barrier, which may in turn increase the chance of infection in the brain of AD patients. The enrichment of normal specific modules also revealed new pathways that may have potential links to AD.

Immune responses were found in AD patients’ brain tissues years ago and vast evidence about it has been accumulated [22, 23]. But how the immune response is triggered is not clear. Recently, a surprising research showed that the accumulation of Amyloid-β as hallmarks of the AD is a defense mechanism and kills infectious agents including viruses or bacteria [47]. More recently, researchers showed that there appeared to be much more bacteria in the AD patients’ brains than normal brains by next-generation sequencing analysis [36]. It is speculated that infections of common bacteria or virus might be a potential cause of AD [36]. Consistent with this notion, our results showed that enriched infections pathways are frequently occurred in AD-specific modules across all three independent datasets we have analyzed, in AD_M9 for dataset GSE5281, AD_M22 for GSE48350, and AD_M25 for Allen Brain dataset. The infections pathways are related to African trypanosomiasis, Malaria, Hepatitis B, and Hepatitis C (see Fig. 3). Moreover, we identified blood-brain barrier tight junction in normal-specific modules, which implies that genes in tight junction pathway lost their coordinated expression patterns in AD brain samples. It is widely acknowledged that blood brain barrier prevents the bacteria or virus from entering the brain [44]. The dysregulated function of tight junction in the blood brain barrier potentially allows the infectious agents entering into the brain [44–46, 48]. Remarkably, in addition to the gain and loss correlation of the specific modules, some of the expression levels of genes in the infectious disease pathways are up-regulated while all of the module genes in tight junction pathway were down-regulated. Our findings not only supported the idea of infection causing AD, but also provided candidate GCN modules and genes in the process.

As we know, biological processes or pathways may be regulated by common upstream regulators. We performed transcription factor analysis of these condition-specific modules and discovered several differentially expressed TFs like TEAD4, STAT1, and JUND that target some of the modules as described in previous sections. The discovery of these key upstream regulators complements the pathways and provides new insights of the mechanism of the disease development. We believe these upstream regulators as the key regulatory genes of the modules could be candidate driver genes of AD. In particular, for AD-specific module AD_M25 enriched in infectious disease related pathways, we identified transcription factor ZFHX3 as a potential driver regulator.

## Conclusions

Our approach identified condition-specific GCN modules using multiple expression datasets from AD and normal multiple brain tissues. Frequently enriched biological processes and pathways provide strong evidences and new insights for AD related pathways and potential AD driver genes. Our results are consistent with recent findings of infection and immune response frequently observed in AD brains, but with more specific insights, which may provide new direction to the mechanism of AD as well as new candidates for therapeutic strategy for AD.

## Additional files

Additional file 1: **Table S1.** Comparison of module genes identified by lmQCM and WGCNA from GSE48350 AD samples. (XLSX 10 kb)

Additional file 2: **Figure S1.** Boxplot of ratios of overlap between modules obtained before and after adding noise to original modules. For 5, 10, and 15% addition of noised data, the ratios of overlaps between modules obtained before and after adding noise to original modules from all modules obtained in three different datasets. (PDF 7 kb)

Additional file 3: **Table S2.** List of 30 AD-specific modules and 31 normal-specific modules. (XLSX 27 kb)

Additional file 4: **Table S3.** Enriched GO terms (*p*-value<0.01) of all condition-specific modules. **Table S4.** Enriched KEGG pathway terms (p-value<0.01) of all condition-specific modules. (XLSX 325 kb)

Additional file 5: **Table S5.** Modules with targets of specific differentially expressed TF enriched in certain pathways. At least two TF targets are in the corresponding enriched pathway. (XLSX 12 kb)

## Abbreviations

AD: Alzheimer’s disease
GCN: Gene co-expression network
lmQCM: Local maximal Quasi-Clique Merger
