# Supplementary figures and images for: Condition-specific gene co-expression network mining identifies key pathways and regulators in the brain tissue of Alzheimer’s disease patients
Source: BMC Med Genomics. 2018 Dec 31;11(Suppl 6):115. doi: 10.1186/s12920-018-0431-1 (PMC6311927; doi:10.1186/s12920-018-0431-1)

# Ratios of Overlap to Original Modules

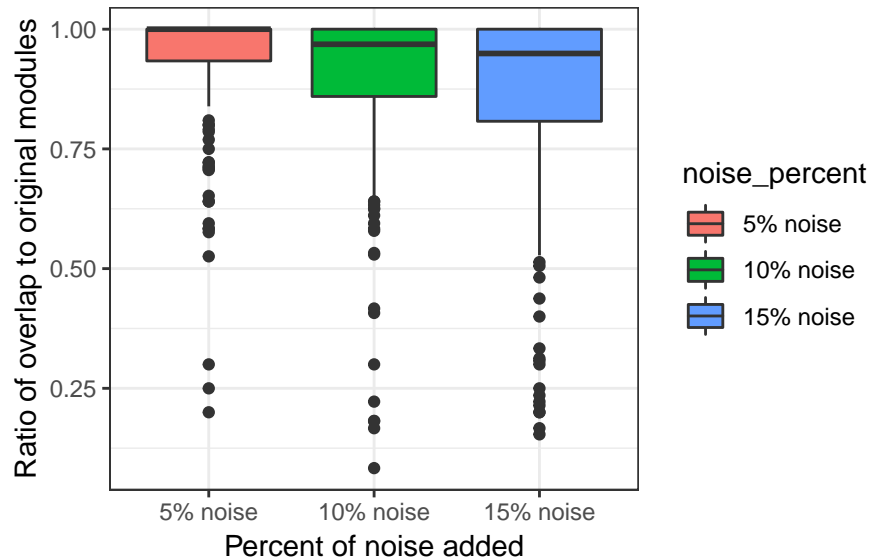

Supplement: Supplementary file 2 — Figure S1. Boxplot of ratios of overlap between modules obtained before and after adding noise to original modules. For 5, 10, and 15% addition of noised data, the ratios of overlaps between modules obtained before and after adding noise to original modules from all modules obtained in three different datasets. (PDF 7 kb) [file 12920_2018_431_MOESM2_ESM.pdf]
